# Enacting person-centred care: a multi-perspective study of practices in clinical encounters for people living with chronic kidney disease

**DOI:** 10.1186/s12882-023-03245-8

**Published:** 2023-06-22

**Authors:** Amie Cotta, Maria Kristiansen

**Affiliations:** 1 Center for IT and Medical technology, Capital Region, Borgervaenget 5, 2100 Copenhagen Ø, Denmark; 2 Department of Public Health, Center for Healthy Aging, University of Copenhagen, Øster Farimagsgade 5, DK 1353 Copenhagen K, Denmark

**Keywords:** Chronic kidney disease, Pre-dialysis, Person-centred care, Shared decision-making

## Abstract

**Background:**

There is growing emphasis on the need for ensuring person-centred care for patients living with chronic kidney disease as this will benefit patients, providers, and healthcare systems alike. Nevertheless, less emphasis is given to how this complex concept is practiced in clinical encounters and how it is experienced by patients. This qualitative multi-perspective study investigates how person-centred care for people living with chronic kidney disease is practiced and experienced by patients in clinical encounters at a nephrological ward at a hospital in the capital region of Denmark.

**Methods:**

The study builds upon qualitative methodologies, including field notes from observations of clinical encounters between clinicians and patients in an out-patient clinic (*n* = ~ 80) and in-person interviews with patients in peritoneal dialysis (*n* = 4). Key themes from field notes and interview transcripts were identified through thematic analysis. Analyses were informed by practice theory.

**Results:**

Findings illustrate that person-centred care is practiced in a relational and situational encounter between patients and clinicians as dialogues about choice of treatment modality, which is shaped by the individual’s life circumstances, preferences, and values. The practice of person-centred care appeared to be complex and interlinked with a range of factors, individual to each patient. We identified three themes of relevance for practices and experiences related to person-centred care: (1) Patients’ perceptions of living with chronic kidney disease. Perceptions differed according to medical history, life situation and prior experiences with treatment in the healthcare system. These patient-related factors were perceived to be important for person-centred care to unfold; (2) Relations between patients and healthcare professionals were important for patients’ experiences of trust and appeared fundamental for the practice and experiences of person-centred care; and (3) Decision-making on treatment modality that is the best fit for each patient’s everyday life, appear to be shaped by the patient’s need for knowledge about treatment modalities and level of self-determination in the decision-making.

**Conclusions:**

The context of clinical encounters influences the practices and experiences of person-centred care, where health policies and lack of embodiment are identified as barriers for providing and experiencing person-centred care.

## Background

A rise in the incidence and prevalence of people suffering from chronic kidney disease (CKD) is observed worldwide. CKD is associated with a high risk of multi-morbidity and a decrease in quality of life while patients undergo complex treatment trajectories [1].

A goal in clinical care is to ensure safety, quality and coordination for people suffering from chronic conditions. This is specifically relevant for chronic kidney disease, since a significant proportion of patients suffer from co-morbidities [2]. An emerging approach to achieve this is through person-centred care. There is no standardized agreed definition of the term in the literature; it is used interchangeably with terms such as ‘patient-centered care’ and ‘whole-person care’ [3]. The concept of person-centred care is well known, but poorly defined and operationalized into practice [4, 5]. In the broader knowledge base, person-centred care comprises of involvement of patients in their own care (individual level), as well as at the program and system levels to ultimately influence the delivery of care that optimizes patient experiences and outcomes. This study focuses on investigating person-centred care at the individual level. This study aims to investigate practices related to person-centred care in a multi-perspective qualitative study to investigate how the concept is operationalized and experienced In the literature, person-centred care is described as an approach to delivering clinical care that is different from the traditional biomedical paradigm due to its enhanced emphasis on the patient’s values, personal choices, and autonomy. Approaches to healthcare services are changing, towards an emphasis on mutual participation of patients and healthcare professionals in clinical activities [6]. Previously, practices have been dominated by clinician-driven activity where new constellations have been introduced. This development consists of a recognition that the health of the patient can be affected by interventions other than merely biomedical approaches [7].

From patients’ perspective, suffering from chronic kidney disease is known to impact quality of life, when in need of dialysis, transplant or experiencing prominent symptoms. International guidelines advise involving patients in decision-making about their treatment modalities and the following care, to secure a well-informed foundation for decisions, based on their personal preferences [6]. This paper conceptualizes person-centred care as a collaboration between patients (relatives) and clinicians in an ongoing and iterative process, where people’s needs, desires, capacities, capabilities and personal or medical situation change and care plans therefore should be flexible [8]. A cornerstone in person-centred care is shared decision-making, which can be described as an approach where healthcare professionals and patients collectively seek to understand the patient’s life circumstances and health to determine what treatment modality is the best option for the patient. In this study, shared decision-making becomes specifically relevant for cases where choice of modalities is discussed. Studies on decision-making, as part of the move towards person-centred care in clinical encounters with patients with chronic kidney disease and clinicians, are limited [1, 9], which call for further studies to unfold how the concept is practiced in clinical encounters [4].

## Methods

### Study design and population

This study takes a qualitative, multi-perspective approach, to investigate how person-centred care is practiced and experienced in clinical encounters at the individual level. We conducted a qualitative study at a nephrological ward at a hospital in the capital region of Denmark during 2019-20. In the initial stages of the study, a panel of patient- and relative representatives were interviewed and discussed experiences related to chronic kidney disease and dialysis. These discussions guided the study design, in terms of methods applied and places to collect data. Negotiation of access was first undertaken through hospital management, accessing health professionals and clinical encounters. Second, due to the General Data Protection Regulation it was not possible to recruit patients for interviews directly. Therefore, nurses recruited relevant patients and established contact with the primary investigator if the patient consented. Initially, shadowing of staff and participation in relevant activities was carried out, in order to secure trustful relations and explore possibilities for access. Even though only a proportion of patients with chronic kidney disease eventually develop chronic kidney failure, the person-centred approach is relevant for both outcomes, but especially for the proportion that eventually need dialysis treatment, and who face a decision on treatment modality.

#### Data collection

Data has been collected through observations and interviews, by the primary investigator. Overview of data collection is shown in Table 1.

**Table 1:** Overview of generated data

Data	Observations in outpatient clinic	Observations in kidney failure clinic	In-person interviews with patients in peritoneal dialysis
Numbers performed	Appr. 80	10	4
Descriptions and examples	Participation in consultations and clinical encounters both in private rooms and hallways. Fieldnotes written during and after consultations.	Participation in consultations and clinical encounters in private rooms. Fieldnotes written during and after consultations.	Audio-recorded semi-structured interviews. All patients were asked to describe their experience in their own words and were presented with findings from observations in order to articulate their own experiences.

### Observations

Clinical encounters in the outpatient clinic (*n* = > 80) and a kidney failure clinic (*n* = 10) were observed by primary researcher, with the purpose of generating insights into the practices, relations and interaction between patients, relatives and health professionals. A majority of observations were performed in the outpatient clinic, which comprised a great variety of patients in terms of age, gender, kidney function, relation to the clinic, medical history, health condition and socioeconomic background. On most days, each doctor in the clinic conducted consultations with approximately 20 patients.

In the kidney failure clinic, patients with an eGFR < 20 were scheduled to prepare for dialysis. At these consultations, both a doctor and nurse attended, and information and planning for dialysis were the centre of focus. Consultations varied from 60 to 90 min and relatives were also frequent participants. The data from observations consisted of written field notes in the native language (Danish, and a few in English).

Data from observations has provided thorough descriptions of what was done and said in all consultations, and a unique opportunity to investigate practices of person-centred care beyond what can be said in interviews, because the complexity of the concept in practice is difficult to define. Therefore, a great emphasis on investigating the concept primarily through observation in real life settings has been chosen, in order to grasp how the concept is operationalized and experienced.

### Interviews

As a supplement to observations, four interviews with patients in peritoneal dialysis were conducted to generate insights and descriptions of experiences related to living with CKD and caregiving. Patients contributed with a retrospective view of their shared decision-making process and interpretation of data from observations, which contributed with articulations and experiences of the themes that emerged from observations. All interviews were transcribed in full in the native language (Danish, and one in English). All participants were offered the opportunity to approve the transcript, and none wished to comment or correct. The interviewees varied in gender (female = 3, male = 1), age (25–70 years), medical history and socio-economic background.

### Presentation of data

Findings from analysis of the data is presented as narratives, all placed within the spectrum of person-centred care. The narratives seek to highlight and exemplify each theme and should not be understood as merely representations of a single theme. The narratives overlap with all identified themes and seek to represent the complexity of person-centred care practices with a focus on each theme in depth.

### Ethics

Formal requirements are fulfilled by following the guidelines and rules of the University of Copenhagen and the General Data Protection Regulation (GDPR). Permission from clinic management was obtained and all participants that were interviewed has provided written consent and were informed about the purpose of research and their rights prior to interview. They were all informed about their right of withdrawing from the research project at any time, and to approve transcripts of the interview, and were informed that the interview was recorded and stored securely according to guidelines. When observing clinical encounters both in the outpatient clinic and the kidney failure clinic, all patients and relatives were asked if they allowed the primary researcher to be present during their consultations.

### Analyses

Analyses were conducted by both authors to facilitate a reflexive dialogue for evaluation of the research, where researchers actively explore alternative interpretations of data [10] and iteratively explore changes in pre-understanding. Analyses to identify themes were informed by practice theory, so that analyses would highlight implicit understanding of routinised and bodily know-how in social interactions [11, 12]. When multiple perspectives on a topic appeared, both authors iteratively reviewed the coding to identify emerging themes and sought to reflect diverse views by including contextual and framing elements, including technologies, material objects and perceptions, to ensure firm grounding in the data [13].

## Results

Through analyses, practices of person-centred care showed complex interactions between patients’ individual needs and healthcare professionals’ experience. We identified three analytical themes that will be presented with examples to elucidate how person-centred care is practiced and experienced in clinical encounters: (1) Patients’ perception; (2) Relations; and (3) Decision-making.

### Theme 1: Perceptions of living with CKD

#### Constructing person-centred care at the end of life

In the following, a patient case is presented where the perception of living with CKD and the complexity of person-centred care stands out. With this perspective, we aim to broaden the understanding of person-centred care where it is shaped by disease perception, patients’ individual wishes, and by the fact that resources in healthcare systems are not always the main challenge to providing the desired care.

At the first consultation of the day, an elderly married couple is scheduled. They are in their 90s and struggle with both their sight and hearing abilities. The husband has in recent years experienced decrease of his kidney function and is now at a stage that the clinic classifies as pre-dialysis. The doctor offers the couple the option of scheduling a longer consultation regarding his approaching kidney failure in the kidney failure clinic, where a nurse and another doctor will present the possibilities for dialysis and other treatment modalities. Both the patient and his wife immediately refuse dialysis treatment. The only thing they are interested in hearing about are possibilities for medical treatment if the husband experiences symptoms from his kidney failure. They do not wish to consult with a new, and to them, unknown doctor to hear about life-prolonging and resourceful treatments that will interfere with their current life. As of the present time, the husband does not experience any symptoms, even though his kidneys are failing. They politely ask whether it is possible to call the doctor and get a prescription if they eventually feel the need. The couple seem very clear in their decision about not to use more time and resources on life-prolonging treatment. The communication between the couple and the doctor is somewhat challenged because of their impaired vision and hearing. The doctor asks several times if they are completely sure about their decision, or if they want to come in again for a consultation. But the couple are very clear about their wishes. It seems obvious that the couple and the doctor have previously talked about where the situation is going, and the couple had some form of silent agreement that medication only for symptoms is the correct choice for them and not dialysis. They explain in a calm and clear manner that they knew this day was coming, and before they leave the doctor’s office, she underlines that it can still be a while before all function of the kidneys is lost. It can still take time, but she wants to be sure that they are offered all options before the kidney failure is too advanced. The couple thank the doctor, saying that they understand and wishing us all a pleasant day. A couple of hours later, I meet the couple again, waiting in the hallway. I ask if they need help with anything, but they tell me that they are waiting for assistance with transport, as they are no longer able to travel by public transport or drive a car. They got up very early in the morning for transport to the hospital in time and will be back home sometime after lunch, all to have only a 15-minute consultation with the doctor. They tell me that they do not mind the waiting time, because it is only once every three months that they go to see the doctors. It would be a very different scenario if it was weekly in dialysis.

This narrative shows how person-centred care is a complex concept to implement systematically in planning for patients’ care. In this case, resources are available in the kidney failure clinic to explain and to begin dialysis treatment, but the circumstances and wishes of the patient determine that person-centred care is not manifested by utilising these resources. Instead, the absence of symptoms and understanding of the development of chronic kidney failure, in combination with the patient’s current life situation, merges with person-centred care when meeting the patient’s wish for minimal interference with his current life by utilising resources from both the patient and the clinic.

Furthermore, we found several instances of patients resisting the focus and refusing life-prolonging treatments. Demand for resources to manage dialysis treatment are massive, for healthcare systems, professionals, and patients, which should result in an informed and shared decision to select quality of life and symptom relief at the end of life. We included the story above to highlight how person-centred care was not only practiced for patients with kidney failure where a lot of time and resources were allocated in a structured process in the kidney failure clinic. This story both relates to the person-centred care in practice, but also to the investigation of what factors are shaping decision-making when kidney failure approaches. Person-centred care became a virtue-directed approach not only to contribute to positive health outcomes, but to respect patients’ wishes. Looking at both individual factors and relational, negotiated factors, creates an insight into how the process towards decision-making is shaped by each contextual contribution, through the overall paradigm’s aim to enable the best possible health and quality of life. Viewing person-centred care through the concept of a spectrum rather than merely as linear planning of the resources of healthcare services, opens the way for a utilisation of resources where they are most needed.

#### Narrative 2: Relations: utilizing resources while facing barriers

The second narrative of a patient case seeks to exemplify how relations between health professionals and patients are fundamental to the ability to practice person-centred care. Resources when needed are crucial for practising person-centred care, however contextual barriers can result in difficulties for all parties.

The next consultation is a follow up with Miss X, who is in her 40s and has a history of alcohol and drug abuse. At the consultation, her appointed social worker from the municipality also attends. The social worker has been with Miss X for 5 years, and attended all the important consultations at the clinic. She is also the contact person for Miss X if the clinic is not able to reach her. The two of them seem to have a very close relationship, and the social worker knows a lot about Miss X’s life circumstances. Presently, Miss X is about to move from her current municipality to the neighbouring one because she needs assistance with maintaining peritoneal dialysis at home. Miss X is sad about the fact that her moving to another assisted housing area, also means that a new social worker will be appointed to her. She is sad that she is basically forced to move, in order to receive the assistance with dialysis that she needs. She will move away from her familiar environment, and what she feels to be her support system. Before the consultation, the nurse warned me that there might be a risk of Miss X not showing up. It had sometimes happened before that she had disappeared for several days and did not show up for the scheduled consultations. At the prior consultation the nurse had agreed with Miss X to start in peritoneal dialysis, because she would not be able to go to the hospital by herself several times a week and be stable. Besides that, her blood flow was very bad so it would be very difficult to operate a well-functioning fistula for hemodialysis. The doctor also checked Miss X’s legs, that were looking blue due to low blood circulation, and she agreed that a fistula would not be a possibility. At the last consultation, the nurse, doctor, social worker, and Miss X had discussed whether the best option was for Miss X to move to similar assisted housing as she had already, in another municipality where it was also possible to get assistance with management of peritoneal dialysis during the night. This is not a health service that is available in her current municipality. They also hoped that if assistance came regularly both at night and in the morning, they could facilitate a more stable routine in her life. The social worker asked how much of a rush they are in to start dialysis, because they are not able to move Miss X into her new housing for a few weeks. The doctor explained that it was very uncertain when Miss X will need dialysis, because her kidney function varies a lot each time they test her. The fluctuations in function can be caused by alcohol or lack of nutrition and water. The nurse asks Miss X if she is looking forward to moving, which she rejects immediately. She is very sad about letting go of her social worker, because she is the one who has helped her the most of all her prior social workers. But on the bright side, she will be moving closer to her boyfriend. The nurse asks Miss X if the couple are together at his apartment, which she confirms, and admits that it is also where she often is when she gets ‘lost’. The nurse points out that when she begins dialysis, she needs to be at home for when the nurse comes at night to start the dialysis. It will not be possible to sleep anywhere else. Miss X is not so thrilled about the message, but mumbles that they will figure it out.

This case exemplifies how close relations and collaboration are crucial in caring for patients that face challenges with handling chronic kidney failure. For health professionals to be familiar with the patient’s life situation and utilise the resources needed to assist with initiating dialysis becomes a determining factor for practicing person-centred care. However, the person-centred care is challenged when political barriers offer different services, dependent on the patient’s place of residence. Left with no other option than to move to the neighbouring municipality, the patient experiences that the service does not prioritise her life situation where she has a close and trustful relation not only to her nurse in the clinic but also with her appointed social worker, that she will now lose. Being vulnerable and dependent on close relations, a move to new housing with an unknown support system, limits the healthcare professionals’ ability to offer person-centred care, because the effects of beginning dialysis are intervening with all aspects of patients’ everyday life. The influence of the structural factors, here health policies, clearly becomes a factor that both shapes the encounter and how person-centred care is facilitated. Including the framing of a clinical encounter opens the way for an understanding that person-centred care is more complex than including the patient as a whole person. The network around the patient, with assisted housing and the fact that she was very content with her social worker, helped her with overcoming both health-related issues and social struggles within her life. But now the circumstances of her health have changed, and she needs dialysis treatment. The framing of the caregiving results in healthcare professionals not being able to meet the needs and preferences of the patient. The care she received at the encounter can still be argued to be person-centred, but the framing of the encounter puts an end to the possibility of meeting the patient’s personal and medical needs in practice. The healthcare system and health policies simply do not favour or prioritise her needs, possibly making her co-morbidities and social struggles more challenging.

#### Narrative 3: Decision-making: integrating medical devices and everyday life

The third theme identified relates to how person-centred care and decision-making are interlinked. Viewing decision-making as part of person-centred care, where decisions of treatment modality become a negotiation through dialogue instead of merely recommendations based on medical factors, offers settings for practicing person-centred care.The practice of person-centred care in clinical settings includes unlocking and engaging with the values and preferences of the patient. 

Mr. X is in his late 50s, and he arrives at the clinic with his wife. He suffers from several co- morbidities, both diabetes and immobility from injuries, and is in a wheelchair. During the last couple of years his kidney function has slowly decreased, and his vital signs are down to a point where the clinic has categorized it as kidney failure, and therefore it is time to start dialysis. The objective of the consultation was to inform Mr. X about the possibilities for dialysis or medical treatment, and eventually to choose what kind of dialysis is best for the patient and to start the process of preparation. Initially Mr. X and his wife address their concerns about their everyday challenges, and say that they cannot overcome further struggles. Every morning, assistance appointed by the municipality comes to help with medication, treatment of wounds, showering and cleaning. The doctor listens and acknowledges their concerns and asks about the symptoms the patient is experiencing. The patient does not recognise any symptoms related to the kidney failure and is mainly concerned about his immobility challenges and ability to manage his pain. The patient’s wife tells the nurse and doctor that they, collectively, have already decided on starting hemodialysis in the hospital, when the time comes. The patient becomes very silent and look down into his lap, and the nurse asks about the thoughts behind the decision. ‘I don’t want any more machines in the house,’ his wife says. A few months earlier, they had received information flyers from another doctor at the clinic, and they realised that they could not live up to the high standards of hygiene, self-care, and storage in their apartment. The patient agrees that they are not able to keep their home clean enough and that he is not able to adhere to the strict timetables of peritoneal dialysis. But at the same time, he is very concerned about going to the hospital three times a week when he is dependent on transportation assistance for getting back and forth to the hospital. He fears that it will take him up to 20 h per week to manage his dialysis, and he is not used to being away from home. He asks if the nurse can help him with getting some timeslots at the hospital that fit his current life the best, but the nurse explains that they cannot meet his wish. Firstly because it is another section of the hospital that manages dialysis but also because most patients wish for the same timeslots in the middle of the day. This message clearly irritates the patient, and he questions whether the dialysis will be worth so much of his already scarce resources.

This case exemplifies how person-centred care is practiced when decision-making is not based on the primary recommendations, but emphasises that the wife and patient have insight into their own life circumstances to assess what treatment modality will eventually be the ‘lesser evil’ for them. Another factor that can be highlighted here is the influence of relatives and the potential effect treatment modality can have. Managing dialysis at home requires many resources, space, and practicalities at home, where assistance in hospital requires many hours and less flexible timetables for patients. Through this story, it becomes clear that what is at stake for the patient and his wife is the suffering related to the practical, behavioural and social challenges, not the biomedical. From a medical perspective, dialysis can improve the biomedical condition of his kidneys’ functions. But for the patient, the dialysis is associated with suffering and with compromises compared to his current life, but he feels he is left with no alternatives. It is about survival. Through his explanation of his worries, he and his wife present insights into the experiences of someone who lives with the condition, and with co-morbidities, that can both influence the experience of symptoms and challenge how the patient experiences the care being centered around his situation.

## Discussion

### Results

The purpose of this study was to gain a deeper, context-specific understanding of how person-centred care is practiced and experienced in the context of clinical encounters between patients with kidney failure and health professionals. This qualitative multi-perspective study adds to the emerging body of literature exploring the complex practices and experiences of patients with chronic kidney disease and health professionals as they engage in decision-making in clinical encounters.

**Fig. 1:**
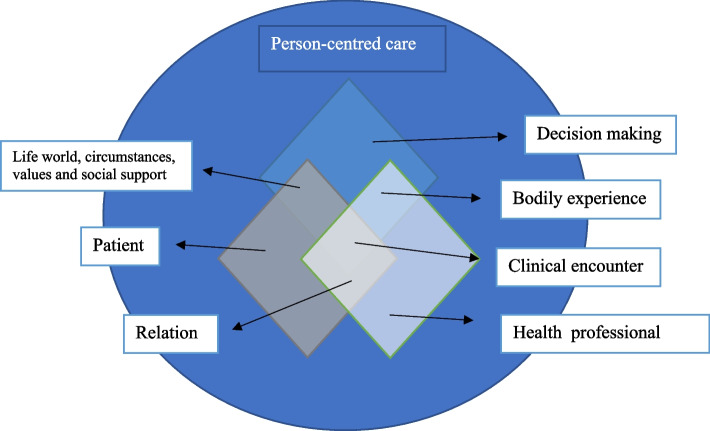
Context of enacting person-centred care. The main elements of the context person-centred care consist of people participating (patients and health professionals, occasionally social support) and the situation with planning the care needed. The size of the elements in the figure do not represent their importance for practicing person-centred care but situate them in the context

Findings in this study highlight that medical history, clinical uncertainty, and practical, behavioural, and social challenges influence patients’ experiences of caregiving. The context that this study investigates and highlight as important to practice person-centred care is illustrated in Fig. 1.

The relations between health professionals and patients have been found to be of importance. Our findings indicate that the organisational context, physical surroundings and health professionals’ ability to emphasise the heterogeneity of patients and continuity of patient-doctor relations are key factors in providing person-centred care, but also challenged in strained healthcare systems. This result underlines how person-centred care as an approach in healthcare systems is defined in clinical encounters by a variety of complex factors, including health policies, health professionals’ skillsets, and patients’ perceptions of disease, their medical history, values and wishes. However given the current contextual changes with growing pressure on sustainable healthcare delivery, lack of continuity and time pressure, affect the possibility to practice person-centred care. In the literature much emphasis is given to shared decision-making and how this concept can facilitate clinical encounters. It is embedded in person-centred care that it is relational and contextually-situated, while our results underline the importance of trustful relations and good communication between patients and health professionals, factors that have been identified as a barrier in other studies [14, 15]. Aiming for structure and developing guidelines or advice can be used as facilitators in conversations about life-changing decisions; but basing a systematic approach that is dependent on people and their emerged skills can be challenged if keypersons or staff change direction in their career and life. The skillset it takes to practice person-centred care is based both on human and professional experience. The settings for the encounter demand resources from health professionals, both in medical and human abilities, which are an element worth taking into account. Practices related to person-centred care have been found to be challenged due to contextual factors in the healthcare system, including pressure on health care systems with fewer resources. Healthcare professionals practice their caregiving for both routine and unexpected needs that appear in clinical encounters, due to the ever-changing personal and medical needs of patients. The constant change of both medical and personal aspects makes healthcare professionals adapt to the new framing of encounters. Person-centred care is a concept that healthcare professionals can actively use as a way of facilitating clinical encounters, but in this study it also appears to be a result of the close relationship between patients, doctors, and nurses. In short, person-centred care can be described as a practice that is informed on the deontological level by virtues, universal in the practice of medicine and individual to the healthcare professional. The practice is manifested through the encounters where the doctor’s and nurse’s skills facilitate the ideal.

In the process of decision-making, factors such as relations to healthcare professionals, life circumstances, perception of illness, technology, time, and bodily experiences have all been identified as important. Decision-making is not a concept that appears to be prominent in patients’ experiences when choosing treatment modality, meaning that if patients were not invited to engage in a dialogue in their decision of treatment modality, they did not question whether the doctor’s recommendation would be the right treatment modality for them. In pre-dialysis, patients face practical, behavioural, and social challenges. Due to the asymptomatic nature of their condition, they experience a lack of bodily experience, where the communication with and relation to the health professional becomes the centre of importance. Some patients feel that they are left translating biomedical terms into their life circumstances. When dialysis is initiated, patients experience a shift in their understanding of illness, and the person-centred care becomes practical through training with an assigned nurse, underlining the need for bodily experience.

In the literature, providing person-centred care is considered to have positive outcomes for both patients and the efficient allocation of resources in the healthcare system [16]. The literature also suggests that there is a need for different types of care for different types of patients, and that patients that face choice on treatment modality for CKD should be provided with unbiased information, making patients’ preferences the leading criterion for decision-making [6, 17]. Insights from this study can benefit further research and considerations in multiple settings. Instead of viewing person-centred care as an approachthat requires many structural, economic, and human resources, it can be viewed as a concept that is shaped through the relational, situated, and contextual settings for clinical encounters.

### Methods

Applying a multi-perspective qualitative study design enables an investigation of person-centred care in clinical encounters. The combination of observations, based on a large number of clinical encounters, that includes a great variety in terms of characteristics of the patients included, along with in-depth interviews with patients, has generated insights into the complexity of structuring a value-based approach to treating patients with chronic kidney disease. To generate further insights into patients’ experiences, further in-depth interviews with groups of patients in other treatment modalities patients could provide important findings, especially emphasising the groups of patients that are struggling with the healthcare services as they are today. People with mental health issues, language barriers, chronic pain or severe co-morbidities seem to struggle with adherence, and experience insufficient caregiving, which calls for further research and initiatives to reach and provide person-centred care. Observations of patients in home settings that practice self-care also have potential for elaborating on how treatment modalities influence patients’ lives. A longitudinal study that follows patients in their process of caregiving could generate insights into the full process of patients experiencing CKD, and can potentially generate valuable insights throughout the patient journey. Data for this study has been generated through observations in real-time and interviews retrospectively. A longitudinal study could benefit from following patients from before their awareness of expected kidney failure, until after a decision on treatment modality and their beginning dialysis. This could give insights, possibly through a narrative study design, and follow the process and person-centred care.

Further investigation of the skillset of health professionals could also add to the understanding of how person-centred care is practiced in clinical encounters, and eventually understanding the needs to practice so from the perspective of healthcare systems and workforces.

## Conclusion

Practices in person-centred care can be viewed as a concept that is an emerged bodily experience, constituted by both clinical expertise and the human skills of the physician. Clinical expertise relates to a combination of physicians’ knowledge of possible treatment modalities and their ability to communicate differences for patients’ lives. Human skills relate to facilitating dialogue with patients and relatives that emphasises each patient’s everyday life, values and wishes. Finally, the context of clinical encounters, and in particular time pressure and physical settings, influence the practices and experiences of person-centred care.

## Data Availability

The datasets generated and/or analysed during the current study are not publicly available due to personal information of participants but are available from the corresponding author on reasonable request.

## References

[1] Levey A, Eckardt K, Tsukamoto Y, Levin A, Coresh J, Rossert J, Zeeuw D, Hostetter T, Lameire N, Eknoyan G (2005). Definition and classification of chronic kidney disease: a position statement from kidney disease: improving global outcomes (KDIGO). Kidney Int.

[2] Lee W, Lee Y, Li L, Ng H, Kuo W, Lin P, Liao Y, Chiou T, Lee C (2018). The number of Comorbidities predicts renal outcomes in patients with stage 3–5 chronic kidney disease. J Clin Med.

[3] American Geriatric Society (2016). Person-centered care: a definition and essential elements. J Am Geriatr Soc.

[4] Byrne A-L, Baldwin A, Harvey C. (2020) “Whose centre is it anyway? defining person-centred care in nursing: An integrative review,” PLOS ONE, 15(3). Available at: 10.1371/journal.pone.0229923. 10.1371/journal.pone.0229923 PMC7064187 32155182

[5] Kunneman M et al. (2021) “Making care fit manifesto,” BMJ Evid Based Med, 28(1):5–6. Available at: 10.1136/bmjebm-2021-111871. 10.1136/bmjebm-2021-111871 PMC9887358 34815303

[6] Covic A, Bammens B, Lobbedez T, Segall L, Heimburger O, van Biesen W, Fouque D, Vanholder R (2010). Educating end-stage renal disease patients on dialysis modality selection. Clinical Kidney Journal.

[7] Gionfriddo M, Leppin A, Brito J, LeBlanc A, Shah N, Montori V (2013). Shared decision-making and comparativeeffectiveness research for patients with chronic conditions: an urgent synergyfor better health. J Comp Effect Res.

[8] Hill N, Fatoba S, Oke J, Hirst J, O’Callaghan C, Lasserson D, Hobbs F (2016). Global prevalence of chronic kidney disease – A systematic review and Meta-analysis. PLoS ONE.

[9] Ghodsian S, Ghafourifard M, Ghahramanian A (2021). Comparison of shared decision making in patients undergoing hemodialysis and peritoneal dialysis for choosing a dialysis modality. BMC Nephrol.

[10] Stige B, Malterud K, Midtgarden T (2009). Toward an agenda for evaluation of qualitative research. Qual Health Res.

[11] Schatzki T (2002). The site of the Social.

[12] Weenink D, Spaargaren G. Practice theories. In: Ritzer G, editor. The Blackwell Encyclopedia of Sociology: Wiley; 2019. p. 1–4. 10.1002/9781405165518.wbeosp125.pub2.

[13] Braun V, Clarke V (2006). Using thematic analysis in psychology. Qual Res Psychol.

[14] Butler CR, Taylor JS, Reese PP, O'Hare AM (2020). Thematic analysis of the medical records of patients evaluated for kidney transplant who did not receive a kidney. BMC Nephrol.

[15] Burns T, Fernandez R, Stephens M (2015). The experiences of adults who are on dialysis and waiting for a renal transplant from a deceased donor: a systematic review. JBI Database System Rev Implement Rep.

[16] Pollak KI, Alexander SC, Tulsky JA, Lyna P, Coffman CJ, Dolor RJ (2011). Physician empathy and listening: associations with patient satisfaction and autonomy. J Am Board Fam Med.

[17] Cramm JM, Leensvaart L, Berghout M, van Exel J (2015). Exploring views on what is important for patient-centred care in end-stage renal disease using Q methodology. BMC Nephrol.

